# Proximity-Based Modalities for Biology and Medicine

**DOI:** 10.1021/acscentsci.3c00395

**Published:** 2023-07-14

**Authors:** Xingui Liu, Alessio Ciulli

**Affiliations:** Centre for Targeted Protein Degradation, Division of Biological Chemistry and Drug Discovery, School of Life Sciences, University of Dundee, 1 James Lindsay Place, Dundee DD1 5JJ, United Kingdom

## Abstract

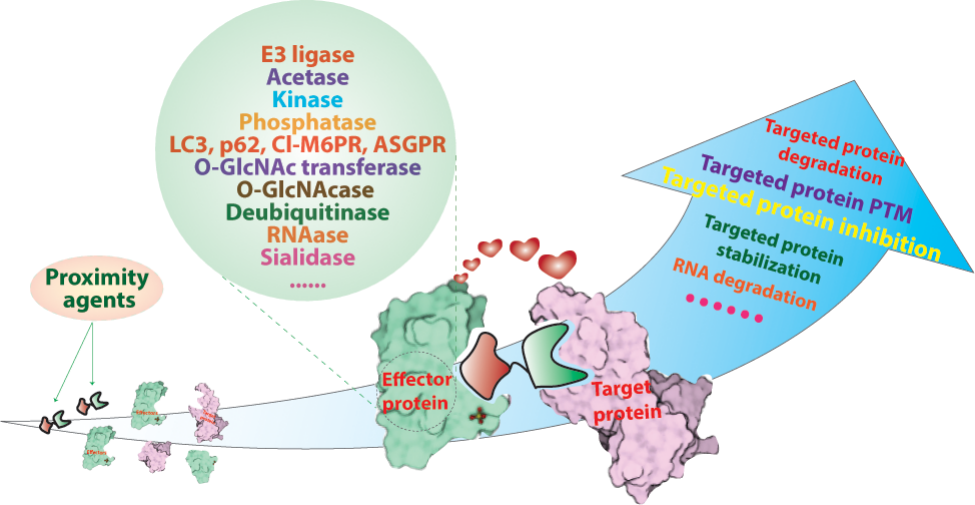

Molecular proximity orchestrates biological function, and blocking existing proximities is an established therapeutic strategy. By contrast, strengthening or creating neoproximity with chemistry enables modulation of biological processes with high selectivity and has the potential to substantially expand the target space. A plethora of proximity-based modalities to target proteins via diverse approaches have recently emerged, opening opportunities for biopharmaceutical innovation. This Outlook outlines the diverse mechanisms and molecules based on induced proximity, including protein degraders, blockers, and stabilizers, inducers of protein post-translational modifications, and agents for cell therapy, and discusses opportunities and challenges that the field must address to mature and unlock translation in biology and medicine.

## Abstract 2

Proximity agents are emerging modalities in biology and drug discovery. They can change the fate of a target and modulate related biological processes by intentionally inducing the proximity between a target and an effector protein.

## Introduction

Proximity and molecular recognition are two fundamental mechanisms for relaying information within and between cells. It was not until the 1990s that artificially inducing proximity was realized to be sufficient to initiate signaling events, when it was found that homodimerizing T cell receptors (TCRs) using antibodies^1,2^ or synthetic dimerizer FK1012^3,4^ could recapitulate TCR signaling in the absence of a T-lymphocyte antigen. Similar approaches were later applied to activate Ras signaling,^5^ death receptor signaling,^6^ and transcription.^7^ These pioneering studies laid the foundation for chemically induced proximity (CIP) in both basic research and drug discovery.

Proteolysis-targeting chimeras (PROTACs) and molecular glue degraders induce proximity between a target protein and a ubiquitin E3 ligase to trigger targeted protein ubiquitination and subsequent degradation.^8−10^ Although PROTACs were conceptualized in the early 2000s,^11^ real-world adoption only occurred when drug-like small-molecule ligands for the E3 ligases VHL and CRBN emerged in the 2010s,^12^ and the field has exploded since 2015.^13^ With around 26 PROTAC degraders (as of June 28, 2023 according to the Beacon database (https://beacon-intelligence.com/)) being advanced into clinical trials, PROTACs have opened a new therapeutic avenue that directs proteins for degradation and have been a leading proximity-based modality in drug discovery.^14^ Bolstered by the success of PROTACs, a plethora of proximity-based modalities have emerged in the last 5 years. These include degraders that work via alternative mechanisms, for example, by hijacking lysosomal proteolytic machineries, and nondegrader molecules that stabilize protein or impact post-translational modifications such as phosphorylation, acetylation, and glycosylation.

Chemically induced proximity holds enormous opportunities to expand the targetable proteome, both intra- and extracellularly, by recruiting suitable effectors to modulate diverse targets, including proteins, nucleic acids, and even organelles. Because of the distinct mechanism via protein dimerization or the formation of ternary complexes, induced proximity is fundamentally distinct from conventional 1:1 antagonists or inhibitors and therefore ushers the development of innovative therapies. However, targeted drug discovery based on proximity beyond small-molecule degraders is still underdeveloped. Early forays by chemical biologists and drug hunters have highlighted important challenges and gaps that the community will need to address to fully enable the potential of the approach.^15,16^ In this Outlook, we discuss major proximity-based modalities according to their mechanisms and offer our perspective on potential opportunities and grand challenges that need to be overcome to unlock this new wave of transformative innovation.

## Various Mechanisms of Proximity-Based Modalities

**Figure 1:**
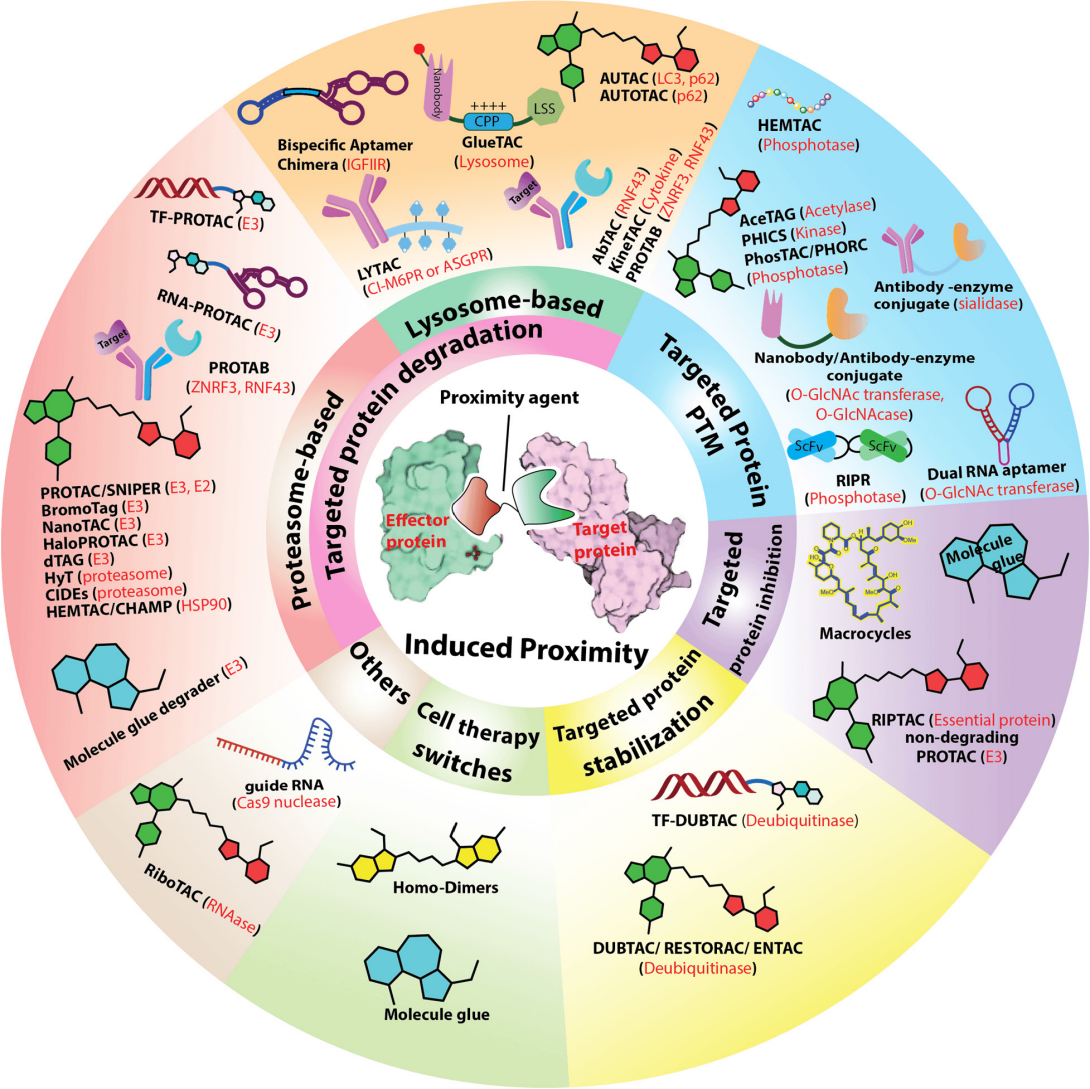
Mechanisms and modalities of induced proximity. Red words in parentheses represent the effector protein(s) recruited by the corresponding modality. RNA-PROTAC: RNA binding proteins targeting proteolysis-targeting chimera. TF-PROTAC: transcription factor targeting proteolysis-targeting chimera. PROTAB. proteolysis-targeting antibodies. PROTAC: proteolysis-targeting chimera. SNIPER: specific and nongenetic inhibitor of apoptosis protein (IAP)-dependent protein erasers. BromoTag, dTAG, HaloPROTACs, auxin-induced degron (AID), and NanoLuc-targeting PROTACs (NanoTACs) are all genetically encoded fusion strategies for targeted protein degradation. HyT: hydrophobic tagging. CIDE: chemical inducers of degradation. HEMTAC: heat shock protein 90 (HSP90)-mediated targeting chimeras. CHAMP: chaperone-mediated protein degradation/degrader. AbTAC: antibody-based PROTACs. KineTAC: cytokine receptor-targeting chimeras. LYTAC: lysosome-targeting chimaeras. AUTAC: autophagy-targeting chimera. AUTOTAC: autophagy-targeting chimera. AceTAC: acetylation tagging system. PHIC: phosphorylation-inducing chimeric small molecules. PhosTAC: phosphorylation-targeting chimeras. PHORC: phosphatase recruitment chimeras. RIPR: receptor inhibition by phosphatase recruitment. RIPTAC: regulated induced proximity-targeting chimera. DUBTAC: deubiquitinase-targeting chimera. ENTAC: enhancement-targeting chimera. TF-DUBTAC: transcription factors targeting deubiquitinase-targeting chimera. RiboTAC: ribonuclease-targeting chimera. Cl-M6PR: cation-independent mannose-6-phosphate receptor. ASGPR: asialoglycoprotein receptor.

**Figure 2:**
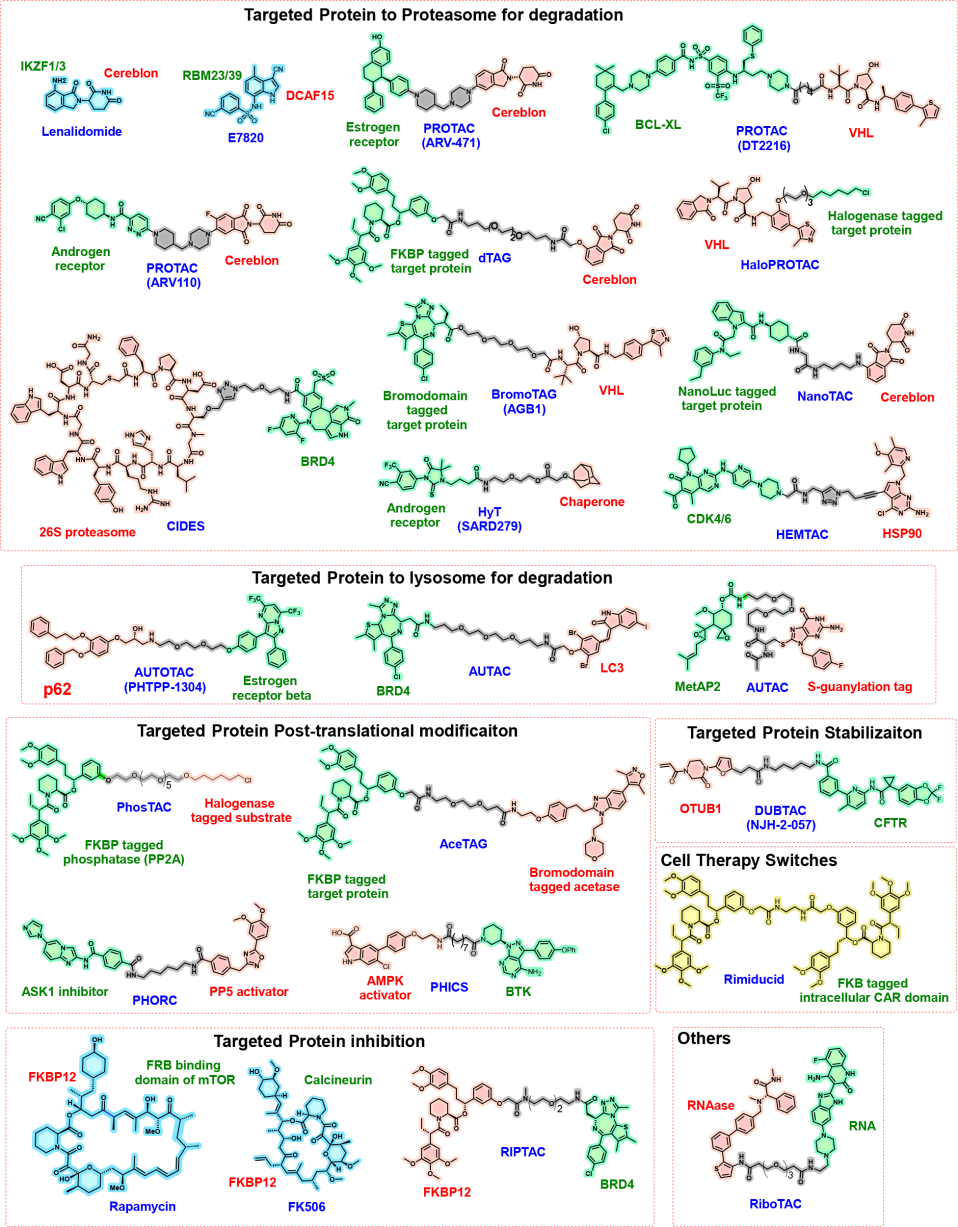
Representative structures of small-molecule proximity agents. The green moiety of the heterobifunctional structures binds to the target protein (in green caption), and the red moiety binds to effector protein (in red caption); the linker parts are shown in gray. The structures in blue are conventionally referred to as molecular glues, with their target proteins and effector proteins shown as green and red captions, respectively. The yellow structure is a homodimer.

Structurally, the existing proximity-based modalities (or proximity agents) can be categorized into monomeric molecules (*e.g.*, molecular glues (MGs)), bifunctional molecules (*e.g.*, PROTACs), or even beyond (*e.g.*, trivalent^17^ or trifunctional molecules).^18^ They cover a wide range of chemical space including small molecules, peptides, proteins, and nucleic acids (Figures 1 and 2). The outcome of induced proximity depends on what target–effector combination is brought together, offering an opportunity to choose the best modality to achieve desired therapeutic effects. Representative effector mechanisms of induced proximity are discussed briefly hereafter.

## Targeted Protein Degradation

### Proteasome-Based Targeted Protein Degradation

MG degraders and PROTACs (or SNIPERs (specific and nongenetic inhibitor of apoptosis protein (IAP)-dependent protein erasers) in cases where inhibitors of an apoptotic protein are recruited as the E3 ligases) are the major and most developed modalities that co-opt E3 ligases to mediate protein degradation via the ubiquitin proteasome pathway. Multiple TAG systems, such as BromoTag,^19^ dTAG,^20^ auxin-inducible degron (AID),^21^ HaloPROTAC,^22,23^ and NanoTAC,^24^ hijack E3 ligases to induce the degradation of engineered fusion proteins inside a cell and hence mostly find applications in biological research. HyT (hydrophobic tagging)^25^ and CIDE (chemical inducers of degradation)^26^ are two of the modalities that are postulated to directly recruit the proteasome for protein degradation, while HEMTAC (heat shock protein 90 (HSP90)-mediated targeting chimeras)^27^ and CHAMP (chaperone-mediated protein degradation/degrader)^28^ are proposed to engage HSP90, a chaperone protein, and therefore recruit multiple E3 ligases indirectly for targeted protein degradation. In the following section, we focus on MG degraders and PROTACs because they are mechanistically and therapeutically the most established (Figure 1).

While the first reported MG degraders can be traced back to plant hormones auxins^29^ and jasmonate,^30,31^ thalidomide is one of the first proximity-based medicines that has been known to induce protein degradation. Its medical use dates back to the 1950s and 1960s when it was prescribed as a sedative to treat morning sickness in pregnant women, leading to the notorious Contergan scandal. However, it was not until 2010 that its target cereblon was identified^32^ and later in 2014 when the MG nature of thalidomide and its derivatives pomalidomide and lenalidomide (collectively known as immunomodulatory drugs) was resolved.^33,34^ By recruiting the neosubstrates of cereblon, such as CK1α and IKZF1/3, to the Cullin 4 RING E3 ligase cereblon (CRL4^CRBN^) and mediating their degradation, these drugs are very efficient against several hematological malignancies among other indications. Additionally, through retrospective studies, aryl sulfonamides including indisulam and E7820 (currently in clinical trials, NCT05024994) were found to be MG degraders of splicing factor RBM39 through the recruitment of the CRL4^DCAF15^ E3 ligase.^35,36^

Discovering newer generations of MG degraders is attractive for drug discovery because of their lower molecular weights and drug-like properties that are more straightforward to optimize compared to larger bifunctional molecules. The ternary complex formation among MG, the target protein, and the E3 ligase is driven by protein–protein interaction (PPI), so high-affinity 1:1 binding to either the target protein or the E3 ligase is not required. However, perhaps for these reasons, discoveries of MG degraders have traditionally occurred more by chance than by design or by optimizing phenotypic activity, as in the case of analogs of immunomodulatory drugs.^37^ Encouragingly, pharmaceutical companies are extensively investigating the field^38^ and more rational and promising strategies are emerging.^39−44^ Some glue firms are introducing artificial intelligence (AI) and AI-powered deep neural networks toward discovery of MG degraders.^38^ Many monovalent molecular glues such as thalidomide and indisulam bind to a specific E3 ligase (CRBN and DCAF15, respectively) and then “glue” neosubstrate proteins. However, increasingly, monovalent MG degraders that bind to the target protein first and then glue via a variety of mechanisms are also emerging. Several MGs are under clinical or preclinical evaluation, including (R)-CR8,^45^ NRX-252114,^44^ and BI-3820.^46^ Beyond monomeric MGs, bifunctional PROTAC-like molecules were recently found to be able to glue the target protein Brd4 to the E3 ligase DCAF16 by intramolecularly bridging two domains of the target protein and anchoring an intrinsic Brd4-DCAF16 protein–protein interaction, without directly engaging DCAF16, leading to very efficient Brd4 degradation^47^ (see refs. (48−49) for related papers published around the same time). Phenotypic screens in cells carrying E3 ligase mutation,^50^ hyponeddylation mutation,^42^ or locking Cullin ligases in an active conformation^39^ have also successfully identified new MG degraders.

**Figure 3:**
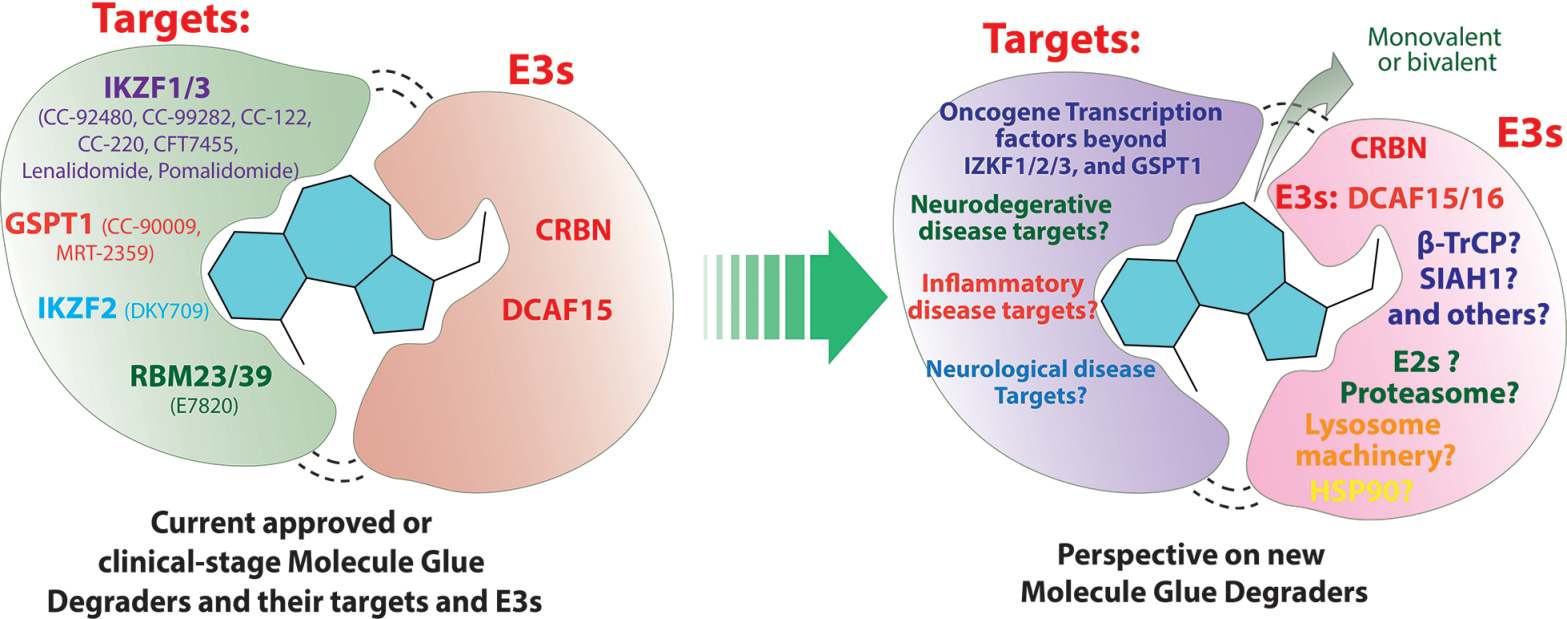
Current approved or clinical-stage molecular glue (MG) degraders and perspective on new MG degraders. Prediction-based data from Drug Hunters.^37^

Ushered by new assays and technologies, we anticipate that the discovery of MG degraders will shift greatly from fortuitous to intentional development. Importantly, increasing evidence supports the emerging concept that MG degraders promote pre-existing E3 ligase-target interactions rather than creating new ones *de novo*.^47,51^ Such interactions are often of weak to intermediate binding affinity and hence inconsequential for effective protein ubiquitination/degradation; therefore, these interactions are challenging to identify or predict using conventional approaches. We anticipate that the development of novel computational and experimental methods to reveal and validate protein–protein interaction pairs will be fundamental to offer a step change in our ability to discover new MG degraders. Recently, proteome-scale induced proximity screens were adopted to identify potential nonphysiological interactors of E3 ligases and deubiquitinases, offering a strategy to address the “effector–target pairing” problem.^52^ For targeted protein degradation, stabilizing interactions with E3 ligases remains the best established mechanism; however, other opportunities may emerge, such as gluing to E2 conjugating enzymes or directly to the proteasome.^26^ Gluing target proteins to other degradation pathways such as the lysosome may also prove effective (Figure 3).

**Figure 4:**
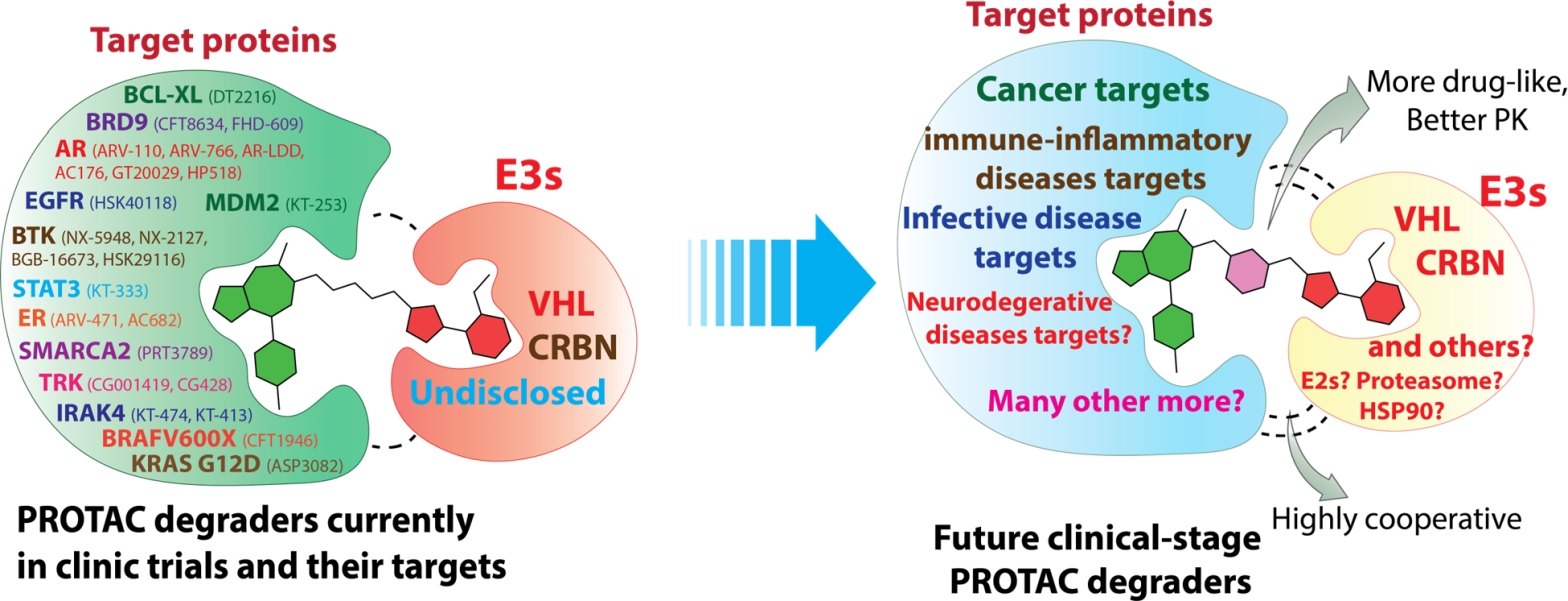
Current PROTAC degraders in clinical studies and perspectives on future clinical-stage PROTAC degraders.

PROTACs are bifunctional molecules designed to induce targeted protein degradation. Because of their modular chemical nature, PROTACs can theoretically be made for any target by tethering a target-binding ligand (of which many abound) with an E3 ligand through a linker. This likely explains the much faster speed of growth of bifunctional PROTACs compared to monovalent MG degraders, with numerous disease-causing proteins been degraded and around 25 PROTAC degraders investigated in clinical trials (Figure 4), making induced protein degradation a paradigm-shifting drug discovery modality.

Compared to traditional occupancy-driven small-molecule inhibitors, PROTAC degraders can chemically knock-down a target protein catalytically and by targeting both the enzymatic and scaffolding functions of a target. The added layer of selectivity by recruiting the E3 ligase and forming a ternary complex can reduce both on-target and off-target toxicity. The enthusiasm and investment in PROTACs from both academia and industry have led to success against many so-called low-hanging fruits, as the majority of the targets being degraded by PROTACs so far are considered druggable or at least ligandable to small molecules. Yet, the design of PROTACs has largely remained an empirical trial-and-error process, limited to E3 ligases that have well-developed ligands, such as von Hippel–Lindau (VHL)^53^ and cereblon.^54^ PROTAC modifications attempting to achieve acceptable drug-like properties (*e.g.*, solubility, metabolic stability, and permeability) and other pharmacokinetic (PK) and pharmacodynamic (PD) properties are often time-consuming and labor-intensive processes compared to molecules of smaller size.

Nevertheless, progress has been made to address these challenges: undruggable targets, including transcription factors (*e.g.*, STAT3 and FOXM1),^55,56^ have been degraded by PROTACs, with STAT3 PROTAC already being advanced to clinical trials (Figure 4); more and more PROTACs are being made orally bioavailable^57−61^ or even blood–brain barrier permeable;^61,62^ and many PROTACs have been dosed to patients in clinical trials showing promising outcomes, reflecting their acceptable PK/PD properties. Expanding the tool box of E3 ligase ligands is becoming an actively pursued research area.^12^ Structure-guided PROTAC design is also enabled through ternary complex structures.^57,63−66^

Moving forward, we anticipate the following challenges associated with developing the next generation of clinical-stage PROTACs:

1. How to degrade untackled disease-causing proteins such as transcription factors and expand to disease areas beyond cancer and inflammatory diseases, **e.g.**, neurodegenerative diseases and infective diseases. Overcoming the blood–brain barrier and co-opting other organismal ubiquitin-proteasome systems^67,68^ are major hurdles for developing PROTACs against neurodegenerative disease targets and anti-infective diseases targets, respectively.
2. How to fast expand the E3 ligase landscape and the E3 ligand toolbox. Cereblon is so far the most prevalent E3 ligase for the first wave of PROTACs in clinical trials. Nonetheless, two VHL-based PROTACs, the Bcl-xL degrader DT2216 and Astellas’s KRAS^G12D^ degrader ASP-3082, have also entered clinical trials.^13,14^ Resistance against CRBN and VHL-based PROTACs develops through mutations and/or downregulation of the ubiquitin ligase machinery,^69−72^ raising concerns about the effectiveness of current PROTACs against highly mutation-prone cancer cells and motivating a need for identifying and developing alternative E3 ligase ligands.
3. How to best explore the chemical space of PROTACs in a more rational and efficient way. Expansion of chemical space to more complex chemistry beyond simple linkers, *e.g.*, covalent chemistries, macrocycles, and conformationally constrained linkers, allows extension to different disease targets and disease areas.
4. How to design highly cooperative PROTACs from low affinity ligands. The lack of tight binders for many undruggable targets, such as transcription factors, means PROTACs for these targets may only be built from low-affinity ligands or even fragments or built on pre-existing intrinsic protein–protein interactions. How to best design degraders by increasing the otherwise too low binary affinity through cooperativity poses an important future challenge for PROTAC design.
5. How to tackle the ADME and PK/PD challenges during the early stage of PROTAC development. Due to the larger molecular sizes and different mechanisms of action, the empirical rules and PK/PD models applied to traditional small-molecule inhibitors are not transferable to PROTACs and therefore may not be used to guide the design of new PROTACs. Nevertheless, the need for orally bioavailable and even blood–brain barrier-permeable PROTAC degraders is increasing as the therapeutic modality expands to disease areas beyond cancer (*e.g.*, neurodegenerative diseases and infective diseases). New empirical rules set specifically for bifunctional molecules,^73,74^ as wells as relevant mechanistic pharmacological PK/PD models^75−79^ that could be further refined by the increasingly available preclinical and clinical data sets of PK/PD studies of PROTACs, are potential solutions to address these challenges.

### Lysosome-Based Targeted Protein Degradation

While MG and PROTAC degraders mainly degrade intracellular proteins through the ubiquitin–proteasome pathway, lysosome-based degraders can target extracellular proteins, transmembrane proteins, intracellular protein aggregates, and even organelles for degradation. These strategies have the potential to greatly expand the degradable proteome and boost the therapeutic reach of targeted protein degradation. Various distinct degradation pathways, including endocytosis, phagocytosis, and autophagy,^80^ can be hijacked for lysosome-based degradation. For example, AUTACs direct target proteins to the autophagy pathway by either labeling them with a degradation tag (guanine derivatives)^81^ or inducing the proximity between target protein and autophagy protein LC3.^82^ Similarly, AUTOTAC recruits target proteins to autophagosome cargo protein p62.^83^ In contrast to AUTAC and AUTOTAC, which are small molecules, LYTAC, PROTAB,^84^ KineTAC,^85^ and AbTAC^86^ are antibody-derived macromolecules. LYTAC is composed of a target protein antibody linked to a ligand of the lysosomal trafficking shuttle (*e.g.*, cation-independent mannose-6-phosphate receptor (CI-M6PR) or asialoglycoprotein receptor (ASGPR)^87^) and therefore can hijack naturally occurring lysosomal trafficking shuttle processes to drag the ligand with its linked extracellular target into lysosomes for degradation. Related strategies include MoDE-As (molecular degraders of extracellular proteins through the ASGPR).^88^ There is also growing evidence that conventional PROTACs target membrane proteins for degradation via recruitment of the intracellular domains of membrane proteins. These PROTACs do not work solely or at all via proteasomal degradation as is the case with cytosolic and nuclear proteins, rather the induced-ubiquitination of the cytosolic domains induce internalization via the lysosomal/autophagy pathway.^89−91^ To circumvent some of these mechanistic limitations for induced degradation of membrane proteins, PROTAB has been proposed as a new modality that recruits cell surface E3 ligases, such as RNF43 and ZNRF3, to induce cell surface protein degradation via both lysosome and proteasome pathways.^84^ Bispecific aptamer chimera is a nucleic acid-based modality that bridges the proximity between membrane-associated proteins and cell-surface lysosome-shuttling receptor (IGFIIR), thereby triggering the lysosomal degradation of membrane proteins.^92^ GlueTAC, a multiple functional modality consisting of a nanobody conjugated with cell-penetrating peptide and lysosome-sorting sequence (CPP-LSS), also targets membrane proteins for degradation (Figure 1).^93^

Lysosome-based targeted protein degradation complements proteasome-based degradation and offers alternative pathways to expand the degradable proteome, but it is still in its infancy. Lysosome is an organelle important for many cellular and physiological functions in addition to protein degradation. It remains to be investigated how susceptible hijacking of lysosomes would be to interference with their normal cellular functions and hence potentially modality-based toxicity. This has been found not to be a problem with proteasomal-based degradation, given the large buffering capacity of the system. The exact mechanisms of many lysosome-based degradation modalities also remain to be fully understood. For example, the molecular mechanism by which *S*-guanylation causes K63 ubiquitination (induced by AUTAC^81^) is not well established.^94^ Their degradation kinetics and potencies are not comparable with those of most well-developed PROTACs and MGs, which may be attributed to the lack of catalytic character or the decomposition of the proximity agents in the acidic and enzymatic lysosome. The potential antigenicity and *in vivo* delivery challenge of macromolecule-based modalities may also be problematic, yet this remains an intense area of development.^95^ To overcome these hurdles, lysosome-based degraders can leverage experiences from PROTAC degraders, such as investigating the structure–activity relationship, in-depth mechanism investigation and developing assays to detect the lysosome function and to monitor each step of the degradation pathway.

## Targeted Protein Inhibition

Cyclosporin, FK506,^96^ and rapamycin^97−99^ were the first generation of proximity-based medicines to be dosed in humans. Retrospective mechanistic studies found that their immunosuppressant activities were attributed to their ability to enhance target protein inhibition by recruiting another binding protein. For example, rapamycin inhibits mTOR much more if FKBP12 is recruited in a ternary complex.^100^ The mechanism of action of these compounds therefore can also be described as that of a MG, albeit of a nondegrading nature.^101^ Other known medicines that also work via this mechanism (nondegrading MG) include paclitaxel,^102^ trametinib,^103^ and anagrelide.^41^ Utilizing PPI to inhibit the enzymatic function or/and scaffolding function of target proteins is an attractive strategy to target challenging proteins and protein complexes, such as transcription factors, and is a promising therapeutic avenue. How to find such MGs beyond serendipity represents a frontier challenge in the field. Proximity-based high-throughput screening (*e.g.*, AlphaLISA, TR-FRET, and NanoBRET) and proximity-based approaches, *e.g.*, BioID and Turbo-ID,^104,105^ are examples of enabling biophysical and cellular technologies for MG discovery. Bifunctional molecules can also act as nondegrading MGs to stabilize PPI through cooperativity and avidity and enhance target inhibition.^106,107^ Choosing the right protein match pair can be challenging. Recently, regulated induced proximity targeting chimera (RIPTAC)^108^ was reported as a new type of bifunctional molecule that can form a cooperative ternary complex between a cancer-specific protein and an essential protein, which abrogates the function of the essential protein and leads to cell death selectively in cancer cells expressing the cancer-specific protein. By leveraging differentially expressed intracellular proteins that are not necessarily tumor drivers, RIPTAC has the potential to widen the therapeutic window of targeting essential proteins (Figure 1).

## Targeted Protein Stabilization

Stabilizing rather than degrading a protein offers a complementary alternative strategy to up-regulate rather than down-regulate target protein levels, opening a new target/disease space. One approach proposed to restore the levels of aberrantly degraded proteins that cause disease and therefore confer therapeutic benefit is to induce the proximity between a deubiquitinase enzyme (DUB) and a target protein using bifunctional molecules called deubiquitinase-targeting chimeras (DUBTACs). The first proof of concept of DUBTACs was reported by Henning and co-workers, in which OTUB1, a DUB that specifically cleaves K48-linked polyubiquitins (degrading ubiquitin chains), was co-opted to stabilize CFTR. Key to their DUBTAC design was the discovery of a covalent ligand against the OTUB1.^109^ This was later harnessed by Liu et al., leading to the development of TF-DUBTACs that can stabilize several tumor suppressor transcription factors, including FOXO3A, p53, and IRF3.^110^

Targeted protein stabilization is an attractive therapeutic strategy that was previously limited to pharmacological chaperones of mutant proteins,^111^ inhibitors of the components in the ubiquitin-proteasome system,^112^ or serendipitous increase of protein levels with small-molecule inhibitors.^113^ By leveraging induced proximity, DUBTACs offer a modular and generalizable approach for rescuing proteins whose destabilization and aberrant degradation lead to diseases.^114^ This technology will likely require expansion to discover other non-orthosteric ligands for DUBs beyond OTUB1 and significant medicinal chemistry optimization to build in the required binding specificity. The human genome encodes more than 100 DUBs. However, effective protein stabilization by DUBTAC might well be restricted to (a) only targets that are substantially ubiquitinated constitutively and (b) only DUBs that cleave degrading ubiquitin chains (*e.g.*, K48 and K11 linkages). In-depth mechanism, function, and structural studies of DUBs are therefore warranted to identify suitable and hijackable target-enzyme space.^114^

## Targeted Protein Post-Translational Modification

**Figure 5:**
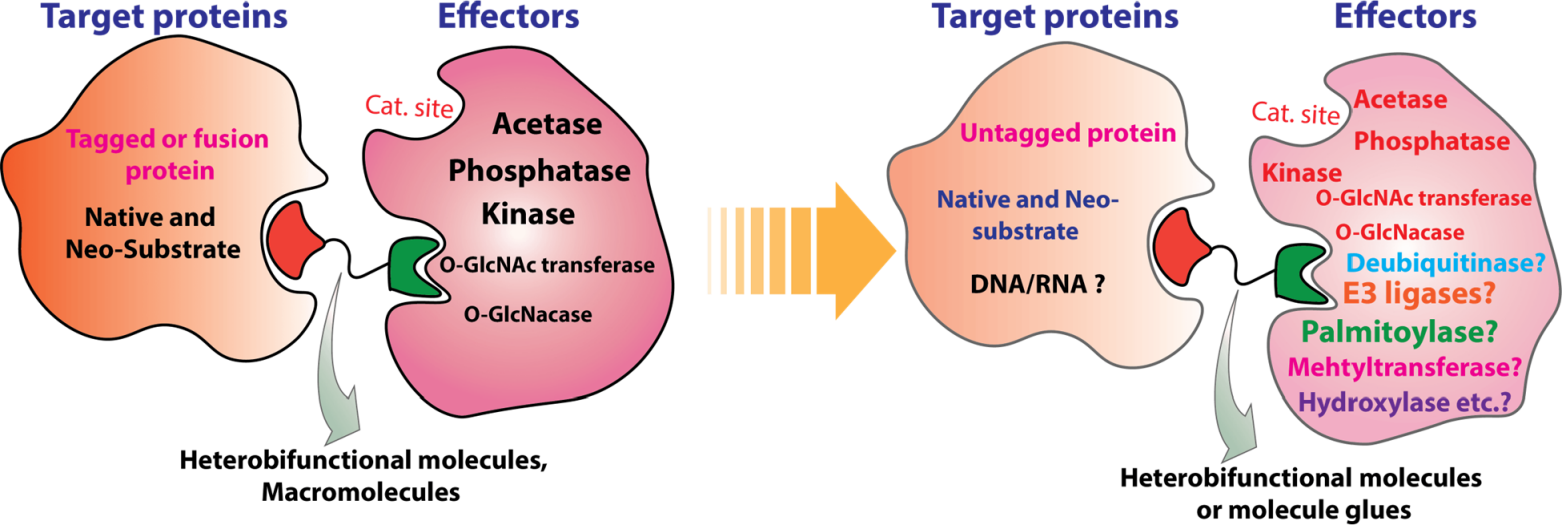
Perspectives on targeted protein post-translational modification.

Post-translational modifications (PTMs), such as phosphorylation/dephosphorylation, acetylation/deacetylation, and ubiquitination/deubiquitination, play critical roles in regulating cellular functions, and aberrant PTM regulations have been implicated in various human diseases.^115^ Conventional ways of targeting PTMs include genetic or chemical modulation of PTM enzymes. More recently, utilizing heterobifunctional molecules to promote the proximity between protein targets and PTM enzymes represents a creative new way to target PTMs. For example, the acetylation tagging system (AceTAG)^116^ was developed to modulate protein acetylation. Phoshorylation-targeting chimeras (PhosTACs),^117,118^ phosphatase recruitment chimeras (PHORCs),^119,150^ receptor inhibition by phosphatase recruitment (RIPR),^120^ and phosphorylation-inducing chimeric small molecules (PHICS)^121^ were designed for precisely controlling protein phosphorylation and dephosphorylation. Other PTMs modulated by proximity-based agents include *O*-GlcNAcylation via fusion of a target-selective nanobody to an *O*-GlcNAc transferase to induce O-GlcNAcylation of endogenous α-synuclein,^122^ as well as nanobodies conjugated to the split O-GlcNAcase eraser enzyme to induce selective deglycosylation of transcription factors c-Jun and c-Fos (Figure 5).^123^ Dual RNA aptamers^124^ have also been proven effective for either glycosylation or deglycosylation of target proteins, while selective removal of sialoglycans from the surface of breast cancer cells was demonstrated using an αHER2 antibody–sialidase conjugate that potentiated the anticancer immune activity of NK cells against cancer cells.^125,126^ (Figure 1)

Compared to traditional PTM-targeting strategies, the proximity-based PTM targeting modality offers on-demand and precise target protein modification without panmodulation effects on other substrates of the corresponding enzymes that install or remove the PTM, referred to as writers or erasers. Considering the diversity of intracellular PTMs, enormous interest will be drawn to the field. Key to the success of this proximity-based modality is the identification of ligands for the writer or eraser that do not inhibit its catalytic activity. Current AceTAGs and PhosTACs rely on fusion protein systems to meet this challenge, in which the heterobifunctional molecules bring the PTM enzymes and protein of interest into proximity by simultaneously targeting the “tag” domain fused with the PTM enzymes and the protein of interest separately. The artificial fusion protein systems are great as chemical biology tools and for proof-of-concept studies. However, to pursue the therapeutic potential of targeted protein PTM, allosteric agonists or nonfunctional ligands against the endogenous writer/eraser are likely required. By tethering allosteric activators of kinases or phosphatase with ligands of the target protein separately, PHICs^121^ and PHORCs^119^ are two new heterobifunctional modalities that rewire the endogenous kinase or phosphatase to precisely phosphorylate or dephosphorylate target proteins, respectively. A study reported by Zhang et al. found that while the ASK1 inhibitor exhibited no effect on MKN45 cells, the PHORCs, composed of ASK1 inhibitor and a phosphatase activator, could reduce p-ASK1^T838^ levels both *in vitro* and *in vivo* and demonstrated anticancer activity on MKN45 cancer cell line and MKN45 xenograft mouse model, suggesting the therapeutic potential of PHORCs as anticancer agents.^119^ Identifying MGs between PTM enzymes and target proteins can potentially be a strategy to bypass ligand discovery for allosteric sites on the writer/eraser.

Besides the aforementioned PTMs that have already been regulated by heterobifunctional modalities, many other PTMs, including ubiquitination, methylation, SUMOylation, hydroxylation, palmitoylation, and even disulfination, also play important roles in regulating signal transduction, protein subcellular localization, PPIs, protein stability, and gene expression. They can theoretically be rewired with bifunctional molecules in a way that is potentially more specific than small-molecule inhibitors (Figure 5).^127^ Moreover, these bifunctional modalities can do both gain of function and loss of function modulations, akin to the PHICs and PHORCs that can phosphorylate and dephosphorylate a protein substrate, respectively. PROTACs and DUBTACs are another pair of complementary modalities for target protein ubiquitination and deubiquitination that act by recruiting the E3 ligase and deubiquitinase separately, leading to protein degradation and stabilization, respectively. However, if nondegrading mono-, multi-, or polyubiquitination chains (*e.g.*, the K63 polyubiquitin chain usually serves as a docking site for PPI to facilitate signal transduction events^127^) are involved in the ubiquitination and deubiquitination process, PROTACs and DUBTACs can also be modalities for tuning post-translational ubiquitination and therefore regulating numerous cellular function and signaling pathways in a precise manner.^128^

The translational potential of the proximity-based PTM targeting modality and how generalizable these mechanisms will be for drug discovery remain to be seen. Most PTMs are tightly regulated and interconnected with each other.^129^ Pairing a druggable PTM with a disease-deregulated protein may be challenging and may depend on the versatility of the PTM-effector and the regulatory network of the target protein. Moreover, such PTM’ed forms of the protein may be present in very low abundance, and their modulation may be too transient to drive a pharmacological response. Together, these observations suggest that careful consideration of the therapeutic concepts behind each proximity-inducing modalities will be important to predict their impact within the complex cellular environment and *in vivo*.

## Cell Therapy Switches

**Figure 6:**
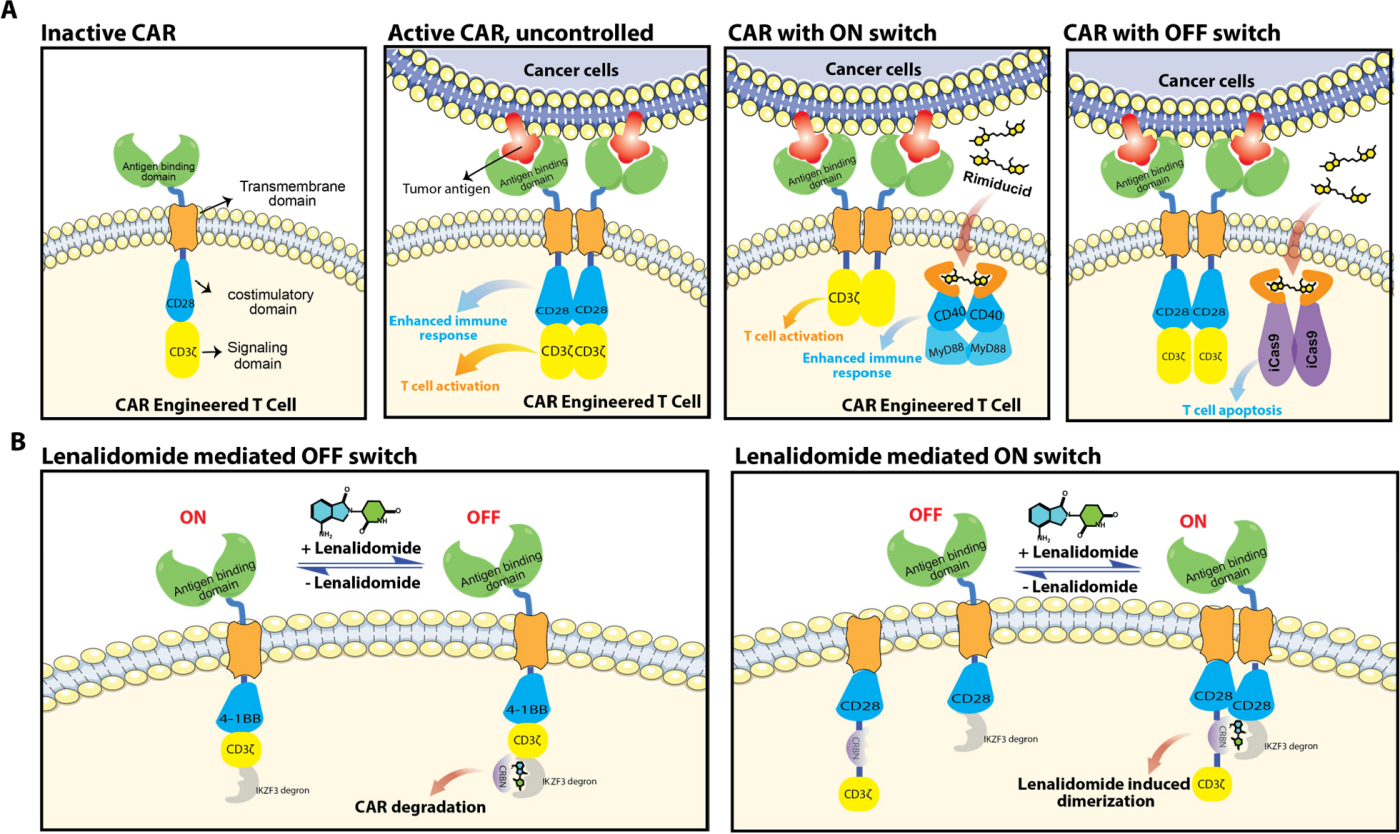
Modalities of advanced cell therapy using proximity agents as ON and OFF switches. (A) CAR-engineered T Cells using Rimiducid as the ON and OFF switch. (B) CAR-engineered T Cells using lenalidomide as the ON and OFF switch.

Cell therapy refers to the administration of viable, often purified, cells to patients to grow, replace, or repair damaged tissue for the treatment of disease. A prominent example of cell therapy involves collecting T-cells from patients and genetic engineering of T-cells to express chimeric antigen receptors (CAR) on the T-cell surface to target a specific type of cancer cells. The genetically modified T-cells are then expanded and administered to patients. The chimeric antigen receptors of CAR-T therapy consist of multiple domains, including the external antigen binding domain, the transmembrane domain, and the intracellular costimulatory domain (*e.g.*, CD28 and 4-1BB) and signaling domain (*e.g.*, CD3ζ) that are responsible for enhancing the immune response and downstream activation and proliferation of T cells, respectively, when these two domains are homodimerized. (Figure 6A). However, insufficient activation of immune response and T cells results into incomplete cancer killing, and hyperactivation of immune response and T cells can lead to toxicities associated with cell therapy, such as graft versus host disease.^130^ Multiple strategies have therefore been developed to modify the chimeric antigen receptor constructs to gain control over the immune response and T cell activation, leading to newer generations of CAR-T technology that are more controllable and safer for patients. Bellicum Pharmaceuticals developed GoCAR-T technology in which the intracellular costimulatory domain is decoupled from chimeric antigen receptors, and rimiducid was introduced to dimerize the costimulatory domain in a proximity-based manner and therefore control the immune response as a ON switch. BPX-601 is such a GoCAR-T technology-based cell therapy studied in a clinical trial (NCT02744287). A proximity-based OFF switch, known as CaspaCIDe, was also developed by Bellicum Pharmaceuticals. The CaspaCIDe switch consists of fusion constructs that tether the FKBP domain with the signaling domain of caspase-9, an enzyme that is part of the apoptotic pathway. Infusion of rimiducid is designed to dimerize the FKBP domains and induce proximity between two signaling domains of caspase-9, which in turn leads to selective apoptosis of the CaspaCIDe-containing T cells and mitigation of the adverse effects associated with T cell hyperactivation. Clinical study of CaspaCIDe-based cell therapy is also ongoing (BPX-603, NCT04650451). Jan et al. developed another CAR-T therapy with ON and OFF switches controlled by lenalidomide (Figure 6B).^131^ The OFF-switch system incorporated an IKZF3 degron tag into the intracellular domains of CAR to enable lenalidomide-induced CAR degradation, whereas the ON switch system comprised a two-component split CAR with CRBN and IKZF3 tagged separately to the two splits, allowing lenalidomide-inducible dimerization.

Although multiple CAR-T cell therapies have been approved by the FDA, the associated toxicities^132^ are one of the major concerns, making CAR-T cell therapy the last resort for many cancer treatments. Temporal control of the CAR-T cell therapy via chemically induced proximity makes the treatment safer and more efficient. Given the engineerability of chimeric antigen receptors and the abundance of monomeric and dimeric chemicals that induce proximity between proteins, the opportunities for developing next-generation CAR-T cell therapy abound. Choosing ligandable constructs that minimally affect the signaling domains of the CAR is critical. Developing proximity-inducible chemicals that are drug-like, safe, and either inert or able to synergize with cell therapy would greatly boost the therapeutic index.

## Other Induced-Proximity Modalities

With induced proximity modality growing at an ever-faster speed, the field is expanding with respect to not only chemical space but also biological space. For example, ribonuclease-targeting chimeras (RiboTACs) recruit ribonucleases to degrade RNA,^133−136^ which expands the target space of induced proximity to RNA and brings the potential to target RNA by recruiting other cellular factors, such as adenosine deaminases, deadenylating enzymes, and terminal U transferase.^137^ CRISPR-Cas9, as one of the most popular gene editing tools, is also a proximity-based technology. The guide RNA is a bifunctional molecule consisting of a crRNA sequence and a tracrRNA sequence that bind specifically to a target DNA sequence and recruit the Cas9 nuclease, respectively, therefore allowing precise DNA cutting by Cas9.^138^ (Figure 1)

## Outlook: Opportunities and Challenges

Proximity-inducing agents co-opt existing cellular machineries to modulate downstream chemistry and signaling, including protein degradation, inhibition, stabilization, localization and post-translational modification, as well as cell therapy control. This Outlook highlights major progress in each of these areas. The meteoric rise of PROTAC and MG degraders and their rapid therapeutic progression with many compounds now being approved drugs or in clinical trials have underpinned a recent surge of new proximity-based modalities. These agents cover a wide range of chemical space, from small molecules to nucleic acids, and disease-relevant biological space, including undruggable transcription factors, intracellular and extracellular proteins, and nucleic acids, and allow for both gain of function and loss of function modulation. Although diverse in their modes of action, they share similar pharmacological and biophysical characteristics, such as event-driven pharmacology and a strong reliance on ternary complex formation. These features give proximity-based agents several advantages compared to occupancy-based agents, such as theoretical lower doses, reduced toxicities, and striking selectivity due to enhanced specificity of molecular recognition within the ternary complex formed. The induced protein–protein interactions, when obtained with high cooperativity and/or avidity, can also greatly boost the potencies of the proximity agents. Cellular localization and relative expression levels of the target and effector protein are important factors to be considered during the design process. In the future, we anticipate that structural and biophysical studies, *e.g.*, using X-ray crystallography and increasingly via cryo-electron microscopy, of the ternary complexes will prove pivotal not only for clarifying modes of action but also for guiding drug design. The multistep mechanisms of action of proximity-inducing agents involving multiple proteins and protein complexes may lead to different mechanism of mutations and drug resistance, as already observed with degraders which can develop resistance via mutations and/or downregulation of the ubiquitin ligase machineries.^69,70,72^ While differentiated in mechanism, novel modalities face often long and tortuous paths to bridge the gap from proof-of-concept to clinically useful agents that benefit patients. In fact, most, if not all of the modalities are still explorative and are at their early stages of development, and caution should be taken before plunging into them, as they may not work solely or at all as intended. For example, multiple PROTAC-like molecules have been reported to work through mechanisms other than those originally anticipated.^47,139,140,89^

The modular and multifunctional nature of most induced-proximity modalities means they will be larger in size than occupancy-based agents. Achieving suitable stability, bioavailability and exposure *in vivo* through medicinal chemistry optimization can be challenging. How to best explore the chemical space, for example, the combination of warhead, linker, and effector ligand, beyond labor intensive and time-consuming combinatorial testing, remains an important goal. Nonetheless, with sound medicinal chemistry optimization of drug-like properties, including the use of more rigid and compact linkers, larger multifunctional molecules can exhibit appropriate pharmacokinetic properties and *in vivo* bioavailability and activities, as amply evidenced by the growing number of clinical-stage and orally bioavailable or even blood–brain barrier-permeable PROTACs.^57−62^ More recently, the application of direct-to-biology (D2B)^141,142^ or related approaches, such as rapid synthesis of PROTACs (Rapid-TAC)^143^ and the “preTACs-cytoblot” platform^144^ to PROTAC synthesis can greatly accelerate exploration of the chemical space and the compound design–make–test cycles. With proximity-inducing agents expanding from small molecules into protein (*e.g.*, AbTAC^86^) and nucleic acids (*e.g.*, dual-specificity RNA aptamers^124^), these biologic-based modalities are potentially associated with antigenicity and will have to face delivery and stability issues *in vivo*, but could also prove tractable.

The dependence of proximity-inducing agents on active effector proteins implies that the effector proteins have to be recruited in a manner that retains and thus effectively redirects their catalytic activity. Protein degraders such as PROTACs take advantage of the complex structures of E3 ligases, where the catalytic sites are typically far away from the substrate binding sites, allowing the development of ligands that bind competitively with substrates but leave the catalytic sites untouched. However, for effector proteins whose substrate binding sites and catalytic sites are close or indeed coincide, this could pose a problem, and recruitment from sites that are remote from catalytic sites is required. Such sites tend to be less conserved than orthosteric sites and may be less ligandable. Novel ligand-finding technologies are required to make these challenging binding sites more tractable. Advances in fragment-based ligand discovery and biophysical and structural technologies to detect, quantify, and locate binding in a more high-throughput manner (*e.g.*, the X-Chem platform at Diamond Light Source^145^) provide many useful starting points for the medicinal chemistry elaboration and development of drug-like ligands.^146,147^ Speeding-up chemistry optimization of the weak-affinity binders that emerge as hits from these screens (*K*_d_ values in the high micromolar to millimolar range) to suitable high-affinity and specific ligands is an important challenge for the field to tackle. DNA-encoded libraries offer large chemical libraries to be screened and benefit from the linkage to genetic barcoding for rapid identification and potential conjugation.^148^ Expansion of chemical space exploration via enabling novel chemistries and diversifying the scaffolds involved, as well as increasing yields and managing side-reactions, will be important areas of focus for encoded technologies. Covalent targeting offers alternative approaches to ligand discoveries, including direct targeting in cells via chemoproteomic approaches. These approaches could potentially be generalized and extended to the discovery of ligands against any target/effector proteins. Caveats and limitations include achieving a suitable balance of reactivity and specificity and limiting off-target effects. Computational approaches and machine learning algorithms will continue to feed new designs and ideas to ligand discovery, and we anticipate the field will become better and better at predicting protein–ligand interactions, binding energies, and enrichment of bona fide binders for proteins.^149^ However, starting from 1:1 binding ligands via one of the aforementioned strategies described above may not always prove tractable. In those cases, identifying MGs between the desired target and a specific effector protein offers an attractive alternative to the design of bifunctional molecules. A targeted search for MGs remains a challenge that we believe will be met through the development of emerging screening technologies and a deeper understanding of the molecular dimerization and protein–protein interaction processes.

In summary, proximity-based agents are leading to a renaissance of induced molecular recognition for biology and medicine that could usher in a drug discovery paradigm with untapped potential. As the field watches with trepidation the progress of the growing pipeline of PROTACs in clinical trials, other proximity agents beyond PROTACs have begun to emerge and reveal new opportunities. The challenges that need to be addressed to enable proximity-inducing molecules to mature into well-established modalities will no doubt push the field toward ever-exciting new developments and discoveries that will unlock new biology and deliver medicines for patients.

## Supporting Information Available

Transparent Peer Review report available (PDF)
