# Supplementary material for: Proximity-Based Modalities for Biology and Medicine
Source: ACS Cent Sci. 2023 Jul 14;9(7):1269–84. doi: 10.1021/acscentsci.3c00395 (PMC10375889; doi:10.1021/acscentsci.3c00395)
Supplement: Supplementary file 1 — oc3c00395_si_001.pdf [file oc3c00395_si_001.pdf]

oc-2023-003959.R1

Name: Peer Review Information for "Proximity-based modalities for biology and medicine"

First Round of Reviewer Comments

Reviewer: 1

Comments to the Author

Dear Authors & Editors,

Thank you very much for giving me the chance to read and review your Outlook “Proximity-based modalities for biology and medicine”. I really liked the manuscript and would like to recommend publishing after some minor revisions. These are mainly concerning adding some more literature, I find important & being a little more critical about the overall potential of these new modalities (although I understand very well how exciting the topic is).

- General:
  - Please give the review another typo-read. There are some typos that can easily be removed.
  - The term “molecule glue” is not as often used as “molecular glue”, which is the more common term. It is also not consistently used one form throughout the whole manuscript.
  - Figure 2: I really like the choice of molecules. However, the current colouring makes it difficult to read the actual chemical structures. As ACS Central Science has most probably a lot of chemists reading it, I would recommend using pastel colours instead to increase readability.
- “Structurally, the existing proximity-based modalities (or proximity agents) can be categorized into monomeric molecules (e.g. molecule glues (MG)) and bifunctional molecules (e.g. PROTACs) or even beyond, e.g. trivalent molecules[14].” I am fine categorizing in glues & bifunctionals. However, the referenced trivalent PROTAC is also a bifunctional molecule with simply three binding sites. I do not consider this fundamentally different as no third function (e.g. a third target) is addressed. A better example would be the following paper: https://doi.org/10.1021/jacs.1c00451. Here, the third functionality – the folate-targeting moiety – addresses a different way of cellular take up.
- “HyT (hydrophobic tagging)[21] and CIDE (chemical inducers of degradation)[22] are two of the modalities that are postulated to directly recruit the proteasome for protein degradation, while HEMTAC (heat shock protein 90 (HSP90)–mediated targeting chimeras)[23] is proposed to engage HSP90 and therefore recruit multiple E3s indirectly for targeted protein degradation.”

    - There was one more direct recruiter to the proteasome using proteasome inhibitors and referred to as “candy approach”. However, the preprint is still not published since 2021.
    - When mentioning HEMTAC, it is probably also fair to mention the CHAMPs approach by Ranok Therapeutics. It has not been published in any peer review journal, but one could cite their patents and they have been actively presenting on several conferences in the recent years. Or is there a reason to leave it out?
- “Beyond monomeric MGs, heterobifunctional PROTAC-like molecules were recently found to be able to glue the target protein Brd4 to the E3 ligase DCAF16 by intramolecularly bridging two domains of the target protein and anchoring an intrinsic Brd4-DCAF16 protein-protein interaction, leading to very efficient Brd4 degradation.[42]”
    - This is indeed a very interesting aspect of the BRD4 degraders. However, it worth mentioning that several groups have been working & publishing on this, not only the Ciulli group 😊. Please also reference
        - https://doi.org/10.1021/acschembio.2c00747
        - https://doi.org/10.1101/2023.02.14.528208
- Clinical stage PROTACs:
    - “With more than 15 PROTAC degraders being advanced into clinical trials, PROTAC has opened a new therapeutic avenue that directs proteins for degradation and has been a leading proximity-based modality in drug discovery” Please give a reference here. You can use your own reference 63 (10.1038/s41571-023-00736-3)
    - “… and more than a dozen of PROTAC degraders have been advanced into clinic trials since, [46]”. Please use the same number. If you only count starting Ph1, I am currently at 22 PROTACs in clinics.
    - In Figure 4, I would change BRAF to BRAFV600X as the mutation is the therapeutic target, moreover KRASG12D (ASP 3082), SMARCA2 (PRT3789) are missing as they already in phase 1, which is also mentioned in the manuscript.
- “How to establish a fully mechanistic pharmacological PK/PD model for PROTACs taking all these factors into account remains challenging, although mathematical models towards this goal are beginning to emerge. [68,69]“
    - I would cite some more literature here, especially the third paper:
        - 10.1074/jbc.RA120.014715
        - 10.1021/acsbiomedchemau.2c00037
        - 10.3390/pharmaceutics15010195
    - In general, I do not see this as a real challenge. Especially in pharma companies, these PK/PD modelling systems are well established, and it just needs a bit more time, experience & data until they will become useful for predicting dosing regimen.

Moreover, up to my knowledge the "hook effect" has not been reported to be observed so far in in vivo experiments, so that the main challenge currently is to achieve sufficient exposure to cover relevant DC50 levels. However, where the hook effect might come into play is when doing toxicity studies. In addition, since MS-techniques become more available and standard in different labs, determining protein-half lives in no longer a challenge, just a to do that is essential for degrader projects.

- "Targeted protein post-translational modification"
    - The authors describe the potential of these modalities as enormous. This view, I do not necessarily share. Of course, there is still a lot to discover and unravel, but it remains to be seen, how generalizable these mechanisms will be for drug discovery. Most PTMs are tightly regulated and interconnected with other PTMs (e.g. other PTMs as pre-activation step) and it will be very hard to find druggable PTM/deregulated protein pairs that can be addressed by a generalizable mechanism of action. Moreover, these deregulated PTM/protein pairs are then usually of rather of low abundance that makes a catalytic MoA or very strong binding necessary. All in all, I am not yet convinced of an enormous potential despite the general scientific interest.
- "Outlook: Opportunities and challenges"
    - What I miss in general the statement that all the described modalities still need ligandable sites, meaning that it does not necessarily expands towards undruggable protein targets. Finding these, remains the challenge.
    - "Although diverse in mode of actions, they share similar pharmacological and biophysical characteristics." → Can you be more precise and name which ones?
    - Especially the newer modalities described using proximity, do not necessarily rely on a catalytic MoA, so that substochiometric target engagement is rather a wish than a reality, even sometimes for PROTACs. Therefore, it remains to be seen how advantageous these MoAs will be as the catalytic nature is needed when designing.
    - Also, and this goes back to the PROTAC challenges No. 4. I do think that cooperativity can solve a lot of problems, but it will not be the answer for all these MoAs. IN this regard, finding strong binders (yes, with the laborious work of a medicinal chemist to do) will most probably stay. We must find smarter&more efficient ways of finding these binders.
    - You also address earlier that it remains challenging how to best explore the relevant chemical space. Of course, AI can help. However, going back to the routes and revive combinatorial chemistry to make degrader discovery more efficient with the help of parallel synthesis that is directly coupled to assay read-outs is currently a very hot topic in pharma and biotechs (Referred to as Direct-to-Biology or Synthesis-to Assay). If you like, you could add this aspect to your review (not a must). I think there are two citable resources
        - JJ paper from last year, 10.1021/acsmedchemlett.2c00124
        - GSK giving several talks on the topic at several conferences

- Another comment, I would like to see when describing these new modalities is the still very explorative character of each described MoA. Currently, for somebody entering the field very recently, it looks as if one could easily use a plug&play approach to get two proteins with different function in proximity and then induce de novo a cellular response. However, more and more reports or unpublished work are also finding that not all the described molecules indeed work as expected. I would give here two examples: One describing a paper that uses AhR as a new E3 Ligase for PROTACs where the molecules were later to be published to rather induce Hydrophobic Tagging, and a recent preprint from Winter & Waldmann finding that a designed AUTAC rather induces DCAF11 recruitment.
    - 10.3390/ph13030034
    - https://chemrxiv.org/engage/chemrxiv/article-details/640b1563b5d5dbe9e8064f48

Reviewer: 2

Comments to the Author

This is a fantastic review written by Ciulli and co-workers that should be published with only minor comments noted below.

Minor points:

1. Figure 2 Rapamycin and FK506 caption colors don't match with structure colors.

2. "By recruiting transcription factors, such as CK1α and IKZF1/3" CK1α is not a transcription factor; please correct.

3. Where do Figures 2 and 5 go in the text?

4. The authors may want to make Figure 2 bigger. It's hard to see the structure due to the size and background color.

5. The citation for RIPR is missing.

6. "heat shock protein 90 (HSP90)–mediated targeting chimeras (HEMTAC), were designed for precisely controlling protein phosphorylation and dephosphorylation" HEMTAC are designed for targeted ubiquitination. Please cite the appropriate reference.

7. Please cite work on toxicities related to CAR-T cell therapy.

8. gRNA can be called bifunctional, but please provide references and applications where this tool has been used to induce proximity (Page 10, lines 40-43).

9. Please provide references for abTAC and RIPR (Page 10, lines 18-20).

Author's Response to Peer Review Comments:

Formatting Needs:

PULL QUOTE (OUTLOOKS + IN FOCUS): We encourage you to select 3 - 4 quotes from your submission that you would like highlighted in your paper. The quotes should be one sentence-long, unique to the submission and not from previously cited work. Please list your quotes at the end of the manuscript file.

The 4 quotes (also listed at the end of the manuscript) we chose are as following:

1. This review outlines the diverse mechanisms and molecules based on induced proximity, including protein degraders, blockers and stabilizers, inducers of protein post-translational modifications, and agents for cell therapy, and discusses opportunities and challenges that the field must address to mature and unlock translation in biology and medicine.
2. Chemically-induced proximity holds enormous opportunities to expand the targetable proteome, both intra- and extra- cellularly, by recruiting suitable effectors to modulate diverse targets, including proteins, nucleic acids and even organelles.
3. The meteoric rise of PROTAC and molecular glue degraders and their rapid therapeutic progression with many compounds now as approved drugs or in clinical trials has underpinned a recent surge of new proximity-based modalities.
4. The dependency of proximity-inducing agents on active effector proteins implies that the effector proteins have to be recruited in a manner that retains and redirects their catalytic activity.

FULL MAILING ADDRESS OF AUTHORS: On the first page of your manuscript, please include the full mailing address of the authors, including a street address and zip code/international code, if possible.

The full mailing address of all the authors is included in the first page of the manuscript as requested.

EMAIL ADDRESS OF CORRESPONDING AUTHOR: Please include the email address of the corresponding author on the first page of the manuscript and the supporting information. Please label as “Email.”

Email address of corresponding author is included on the first page of the manuscript.

AUTHOR AFFILIATION LABELS: Author names and affiliations should be present at the beginning of the manuscript underneath the title. Each affiliation should have a label (for example 1, 2, 3 etc), and the labels should be present by the authors’ names.

Each author has been labeled and affiliation assigned together with the mailing address.

IMPROPER TOC FILE DESIGNATION: Please re-designate as “Graphic for Manuscript.” Visit

http://pubsapp.acs.org/paragonplus/submission/toc_abstract_graphics_guidelines.pdf? for more information.
TOC file has been renamed to "Graphic for manuscript". And also placed at the end of manuscript.

SYNOPSIS MISSING: The synopsis should be no more than 200 characters (including spaces) and should reasonably correlate with the TOC graphic. The synopsis is intended to explain the importance of the article to a broader readership across the sciences. Please place your synopsis in the manuscript file after the TOC graphic.

A synopsis is provided at the end of the manuscript after the TOC graphic.

Reviewer(s)' Comments to Author:

Reviewer: 1

Recommendation: Publish in ACS Central Science after minor revisions noted.

Comments:
I added a docx file with my points (**comments already pasted below and addressed point by point**).

Additional Questions:
Quality of experimental data, technical rigor: High

Significance to chemistry researchers in this and related fields: Top 5%

Broad interest to other researchers: High

Novelty: Top 5%

Is this research study suitable for media coverage or a First Reactions (a News & Views piece in the journal)?: No

Dear Authors & Editors,
Thank you very much for giving me the chance to read and review your Outlook "Proximity-based modalities for biology and medicine". I really liked the manuscript and would like to recommend publishing after some minor revisions. These are mainly concerning adding some more literature, I find important & being a little more critical about the overall

potential of these new modalities (although I understand very well how exciting the topic is).

- General:

Please give the review another typo-read. There are some typos that can easily be removed.

Thanks for the suggestions, the review has been typo-proof read and typos are corrected as track-change.

The term "molecule glue" is not as often used as "molecular glue", which is the more common term. It is also not consistently used one form throughout the whole manuscript.

We thank the reviewer for pointing this out, we have unified the term as "molecular glue" (MG) throughout the manuscript.

Figure 2: I really like the choice of molecules. However, the current colouring makes it difficult to read the actual chemical structures. As ACS Central Science has most probably a lot of chemists reading it, I would recommend using pastel colours instead to increase readability.

Thanks for the suggestions, the coloring style has been modified to improve readability as suggested.

"Structurally, the existing proximity-based modalities (or proximity agents) can be categorized into monomeric molecules (e.g. molecule glues (MG)) and bifunctional molecules (e.g. PROTACs) or even beyond, e.g. trivalent molecules[14]." I am fine categorizing in glues & bifunctionals. However, the referenced trivalent PROTAC is also a bifunctional molecule with simply three binding sites. I do not consider this fundamentally different as no third function (e.g. a third target) is addressed. A better example would be the following paper: https://doi.org/10.1021/jacs.1c00451. Here, the third functionality – the folate-targeting moiety – addresses a different way of cellular take up.

We have included a citation to the suggested paper (reference 18).

"HyT (hydrophobic tagging)[21] and CIDE (chemical inducers of degradation)[22] are two of the modalities that are postulated to directly recruit the proteasome for protein degradation, while HEMTAC (heat shock protein 90 (HSP90)–mediated targeting

chimeras)[23] is proposed to engage HSP90 and therefore recruit multiple E3s indirectly for targeted protein degradation." There was one more direct recruiter to the proteasome using proteasome inhibitors and referred to as "candy approach". However, the preprint is still not published since 2021. When mentioning HEMTAC, it is probably also fair to mention the CHAMPs approach by Ranok Therapeutics. It has not been published in any peer review journal, but one could cite their patents and they have been actively presenting on several conferences in the recent years. Or is there a reason to leave it out?

We thank the reviewer for bringing this up, we have added CHAMPs approach in both Figure 1 and the main text referred by the reviewer. Ranok therapeutics' patent is also cited (reference 28) as suggested.

"Beyond monomeric MGs, heterobifunctional PROTAC-like molecules were recently found to be able to glue the target protein Brd4 to the E3 ligase DCAF16 by intramolecularly bridging two domains of the target protein and anchoring an intrinsic Brd4-DCAF16 protein-protein interaction, leading to very efficient Brd4 degradation.[42]" This is indeed a very interesting aspect of the BRD4 degraders. However, it worth mentioning that several groups have been working & publishing on this, not only the Ciulli group ☺. Please also reference https://doi.org/10.1021/acschembio.2c00747

https://doi.org/10.1101/2023.02.14.528208

We now cite the two papers (references 48 and 49) as suggested.

Clinical stage PROTACs: "With more than 15 PROTAC degraders being advanced into clinical trials, PROTAC has opened a new therapeutic avenue that directs proteins for degradation and has been a leading proximity-based modality in drug discovery" Please give a reference here. You can use your own reference 63 (10.1038/s41571-023-00736-3)

The suggested reference (now reference 14) is cited at this point. The number of clinical-stage PROTAC degraders have also been updated.

"… and more than a dozen of PROTAC degraders have been advanced into clinic trials since, [46]". Please use the same number. If you only count starting Ph1, I am currently at 22 PROTACs in clinics.

Thanks for pointing this out. We have checked the Beacon database on the number of PROTAC degraders in clinical trials and arrived at 25 as of 16 May 2023. We updated the numbers accordingly in the main text.

In Figure 4, I would change BRAF to BRAFV600X as the mutation is the therapeutic

target, moreover KRASG12D (ASP 3082), SMARCA2 (PRT3789) are missing as they already in phase 1, which is also mentioned in the manuscript.

Thanks for the suggestions, we have corrected and/or added these suggested information and added other clinical stage PROTACs into Figure 4.

"How to establish a fully mechanistic pharmacological PK/PD model for PROTACs taking all these factors into account remains challenging, although mathematical models towards this goal are beginning to emerge. [68,69]" I would cite some more literature here, especially the third paper:

- 10.1074/jbc.RA120.014715
- 10.1021/acsbiomedchemau.2c00037
- 10.3390/pharmaceutics15010195

In general, I do not see this as a real challenge. Especially in pharma companies, these PK/PD modelling systems are well established, and it just needs a bit more time, experience & data until they will become useful for predicting dosing regimen. Moreover, up to my knowledge the "hook effect" has not been reported to be observed so far in in vivo experiments, so that the main challenge currently is to achieve sufficient exposure to cover relevant DC50 levels. However, where the hook effect might come into play is when doing toxicity studies. In addition, since MS-techniques become more available and standard in different labs, determining protein-half lives in no longer a challenge, just a to do that is essential for degrader projects.

We thank the reviewer for recommending these papers, they have been cited as suggested (references 77, 78, and 79), and we have reworded this part as shown in the track-changed supplement.

"Targeted protein post-translational modification"

The authors describe the potential of these modalities as enormous. This view, I do not necessarily share. Of course, there is still a lot to discover and unravel, but it remains to be seen, how generalizable these mechanisms will be for drug discovery. Most PTMs are tightly regulated and interconnected with other PTMs (e.g. other PTMs as pre-activation step) and it will be very hard to find druggable PTM/deregulated protein pairs that can be addressed by a generalizable mechanism

of action. Moreover, these deregulated PTM/protein pairs are then usually of rather of low abundance that makes a catalytic MoA or very strong binding necessary. All in all, I am not yet convinced of an enormous potential despite the general scientific interest.

We appreciate the critical comments from the reviewer. We have reworded the sentence and incorporated some of the reviewer's critical points into this part of the manuscript.

"Outlook: Opportunities and challenges"

What I miss in general the statement that all the described modalities still need ligandable sites, meaning that it does not necessarily expands towards undruggable protein targets. Finding these, remains the challenge. "Although diverse in mode of actions, they share similar pharmacological and biophysical characteristics." □ Can you be more precise and name which ones?

Thanks for pointing this out, we have modified this sentence to "*Although diverse in mode of actions, they share similar pharmacological and biophysical characteristics, such as event-driven pharmacology and strong reliance on ternary complex formation*", to be more precise.

Especially the newer modalities described using proximity, do not necessarily rely on a catalytic MoA, so that substochiometric target engagement is rather a wish than a reality, even sometimes for PROTACs. Therefore, it remains to be seen how advantageous these MoAs will be as the catalytic nature is needed when designing. Also, and this goes back to the PROTAC challenges No. 4. I do think that cooperativity can solve a lot of problems, but it will not be the answer for all these MoAs. IN this regard, finding strong binders (yes, with the laborious work of a medicinal chemist to do) will most probably stay. We must find smarter&more efficient ways of finding these binders. You also address earlier that it remains challenging how to best explore the relevant chemical space. Of course, AI can help. However, going back to the routes and revive combinatorial chemistry to make degrader discovery more efficient with the help of parallel synthesis that is directly coupled to assay read-outs is currently a very hot topic in pharma and biotechs (Referred to as Direct-to-Biology or Synthesis-to Assay). If you like, you could add this aspect to your review (not a must). I think there are two citable resources

□ JJ paper from last year, 10.1021/acsmedchemlett.2c00124

□ GSK giving several talks on the topic at several conferences

We thank the reviewer for their helpful insight and for bringing the "Direct-to-Biology" or "Synthesis-to-Assay" to our attention. We have added this aspect to our manuscript and cited related papers (references 141, 142, 143, and 144), including the one (reference 141) suggested by the reviewer.

Another comment, I would like to see when describing these new modalities is the still very explorative character of each described MoA. Currently, for somebody entering the field very recently, it looks as if one could easily use a plug&play approach to get two proteins with different function in proximity and then induce de novo a cellular response. However, more and more reports or unpublished work are also finding that not all the described molecules indeed work as expected. I would give here two examples: One describing a paper that uses AhR as a new E3 Ligase for PROTACs where the molecules were later to be published to rather induce Hydrophobic Tagging, and a recent preprint from Winter & Waldmann finding that a designed AUTAC rather induces DCAF11 recruitment.

- 10.3390/ph13030034
- https://chemrxiv.org/engage/chemrxiv/article-details/640b1563b5d5dbe9e8064f48

We thank the reviewer for these suggestions, and we agree with the reviewer that most of the modalities are still explorative and are at their early stages of development. They may not work as intended, as evidenced by reports and from our own experience, as well as unpublished work, which offer cautionary tales for people new to the field. We have included this aspect in the "Outlook: opportunities and challenges" part and cited the paper suggested (references 139 and 140), in addition to one other related paper (reference 89).

Reviewer: 2

Recommendation: Publish in ACS Central Science after minor revisions noted.

Comments:
This is a fantastic review written by Ciulli and co-workers that should be published with only minor comments noted below.

Minor points:
1. Figure 2 Rapamycin and FK506 caption colors don't match with structure colors.

Thanks for pointing this out, we have intentionally shown the structures of molecular glues in blue to differentiate them from bifunctional molecules. The green caption shows target proteins and red caption shows target proteins of the molecular glues to be consistent with bifunctional molecules.

2. “By recruiting transcription factors, such as CK1α and IKZF1/3” CK1α is not a transcription factor; please correct.

This sentence has been corrected to “By recruiting the neo-substrates of cereblon, such as CK1α and IKZF1/3”

3. Where do Figures 2 and 5 go in the text?

Thanks for spotting this. Figures 2 and 5 have been inserted at proper locations in the text.

4. The authors may want to make Figure 2 bigger. It’s hard to see the structure due to the size and background color.

The colors of the structures have been modified for better visibility. We prefer all the structures are shown in one figure and one page for comparison, that’s the largest possible size.

5. The citation for RIPR is missing.

The citation for RIPR (reference 120) has been added.

6. “heat shock protein 90 (HSP90)–mediated targeting chimeras (HEMTAC), were designed for precisely controlling protein phosphorylation and dephosphorylation” HEMTAC are designed for targeted ubiquitination. Please cite the appropriate reference.

Thank the reviewer for pointing this mistake out, we have corrected this sentence as track change in the manuscript. Reference for HEMTAC is also cited (reference 27).

7. Please cite work on toxicities related to CAR-T cell therapy.

References 130 and 132 talking about the toxicities of CAR-T cell therapy are cited in the manuscript.

8. gRNA can be called bifunctional, but please provide references and applications where this tool has been used to induce proximity (Page 10, lines 40-43).

A paper (reference 138) about the history and application of gRNA was cited as advised.

9. Please provide references for abTAC and RIPR (Page 10, lines 18-20).

Citation (reference 86) for abTAC was added. However, RIPR was not the right modality to be mentioned here and has been replaced with dual-specificity RNA aptamers, for which reference 124 was referred.

Additional Questions:
Quality of experimental data, technical rigor: Top 5%

Significance to chemistry researchers in this and related fields: Top 5%

Broad interest to other researchers: Top 5%

Novelty: Top 5%

Is this research study suitable for media coverage or a First Reactions (a News & Views piece in the journal)?: No
